# Association between serum levels of insulin‐like growth factor‐1, bioavailable testosterone, and pathologic Gleason score

**DOI:** 10.1002/cam4.1681

**Published:** 2018-07-10

**Authors:** Myong Kim, Jong Won Kim, Jong Keun Kim, Sang Mi Lee, Cheryn Song, In Gab Jeong, Jun Hyuk Hong, Choung‐Soo Kim, Hanjong Ahn

**Affiliations:** ^1^ Department of Urology Asan Medical Center University of Ulsan College of Medicine Seoul Korea; ^2^ Department of Urology Korea Cancer Center Hospital Seoul Korea

**Keywords:** bioavailable testosterone, Gleason score, insulin‐like growth factor‐1, pathology, prostate cancer

## Abstract

**Background:**

We evaluated the association between serum levels of insulin‐like growth factor‐1 (IGF‐1), bioavailable testosterone, and surgical Gleason score (GS).

**Methods:**

We analyzed 793 patients who underwent radical prostatectomy and 272 men with negative prostate biopsy. Serum levels of IGF‐1 and testosterone were measured before surgery or biopsy.

**Results:**

The mean IGF‐1 levels of prostate cancer patients and men with a negative biopsy were 143.8 and 118.9 ng/mL, respectively (*P *<* *0.001). Men with high serum IGF‐1 were more likely to have prostate cancer (highest vs lowest quartile, odds ratio [OR] = 3.35; *P*_trend_ < 0.001). However, among men with prostate cancer, the mean IGF‐1 levels of those with low (GS ≤ 6), intermediate (GS = 7), and high surgical GS (GS ≥8) were 151.7, 144.1, and 132.9 ng/mL, respectively (*P *<* *0.001). Using quartile analysis, high serum IGF‐1 levels were shown to be associated with a low risk of high surgical GS (OR = 0.464; *P*_trend_ = 0.006). Serum bioavailable testosterone concentration was positively correlated with serum IGF‐1 level (*r *=* *0.157, *P *<* *0.001). High bioavailable testosterone level was also associated with a low risk of high surgical GS in patients without diabetes mellitus (OR = 0.569; *P*_trend_ = 0.040). Among men with biopsy GS ≤ 3 + 4 (*n *=* *460), upgrading to high surgical GS was more frequent in patients with low IGF‐1 level (≤116.0 ng/mL; 9.9%) or low bioavailable testosterone level (≤0.85 ng/mL; 9.3%) than in patients with normal IGF‐1 and bioavailable testosterone levels (2.6%; *P *=* *0.004).

**Conclusions:**

Serum levels of IGF‐1 and bioavailable testosterone show inverse associations with high surgical GS. This suggests that high‐grade prostate cancer develops independently of these two substances.

## 1 INTRODUCTION

Serum insulin‐like growth factors (IGFs) have mitogenic and anti‐apoptotic effects on normal and transformed prostatic epithelial cells.^1^, ^2^, ^3^ Of these, IGF‐1 mainly originates in the liver and shows low inter‐individual variability in its circulating levels.^4^ Binding of ligand to the IGF‐1 receptor (IGF1R) leads to the phosphorylation of Src homology 2 domain‐containing (SHC)‐transforming protein and subsequent activation of the Ras pathway, and phosphorylation of insulin receptor substrate (IRS)‐1 protein, which induces the activation of the phosphoinositide 3‐kinase (PI3K)/protein kinase B (AKT) pathway.^5^ The relationship between serum IGF‐1 level and prostate cancer is of interest, because previous studies have suggested that aspects of energy metabolism and balance are associated with the incidence of prostate cancer,^6^ and the effect of environmental factors on prostate cancer risk may be mediated by differences in the concentrations of substances including testosterone and prostate‐specific antigen (PSA).^7^

Previous epidemiologic studies report that high circulating IGF‐1 is associated with a higher risk of prostate cancer.^8^, ^9^ Serial reports from the Health Professionals Follow‐up Study (HPFS), a large prospective cohort study comprising 51 529 male health professionals from the United States, aged 40‐75 years at enrollment in 1986, demonstrate that circulating IGF‐1 level is positively associated with low‐grade, but not with high‐grade, prostate cancer.^10^, ^11^, ^12^ Associations between serum IGF‐1 level and low‐grade prostate cancer have also been shown by a pooled analysis of 12 prospective studies,^13^ and the results of other large prospective cohort studies, such as the Physicians’ Health Study (PHS) 14 and the European Prospective Investigation into Cancer and Nutrition (EPIC) study.^15^ These findings imply that there are stronger effects of IGF‐1 on the development of low‐grade prostate cancer than on the development of high‐grade disease. However, a convincing biological mechanism that can explain this observation has not been identified, although tumor growth or development in poorly differentiated cancers may be more autonomous.

Since the demonstration of hormonal responsiveness in 1941 by Huggins et al.^16^ the relationship between serum testosterone and prostate cancer has been thought to be in the form of “fuel for a fire”.^17^ However, the association between serum testosterone and prostate cancer has come under greater scrutiny over the last decade, and has been shown to be similar to that between serum IGF‐1 level and prostate cancer.^18^, ^19^, ^20^, ^21^, ^22^, ^23^ Although higher levels of serum testosterone (or bioavailable testosterone) are associated with a greater risk of prostate cancer,^18^, ^19^ these levels showed an inverse association with tumor grades.^19^, ^20^, ^21^, ^22^, ^23^ An androgen‐depleted environment, with low serum testosterone levels, may foster the development of high‐grade prostate cancer by selecting for molecular events that induce aggressive tumor characteristics.^22^ This mechanism may account for the results of clinical studies in which low testosterone level is associated with high‐grade prostate cancer.^23^

We hypothesized that low IGF‐1 levels might be associated with high‐grade disease, because IGF‐1 induces prostate cancer development *via* the androgen axis or has similar effects to testosterone. To test this hypothesis, we assessed the relationships among the serum levels of IGF‐1, testosterone, and bioavailable testosterone, and the Gleason score (GS), in radical prostatectomy (RP) specimens, using samples collected from RP cohorts, in which the serum IGF‐1 and other hormone levels were prospectively measured.

## 2 MATERIALS AND METHODS

### 2.1 Patients

The study protocol was approved by our institutional review board (no. S2016‐2002‐0001). The need for informed consent was waived by the institutional review board owing to the minimal risk of harm. All the individual identifiers were anonymized and analyzed. The study population comprised patients who underwent RP between 2011 and 2016 at our institution. The serum IGF‐1, IGF binding protein‐3 (IGFBP‐3), testosterone, albumin, and sex hormone‐binding globulin (SHBG) levels, which affect the bioactivity of IGF‐1^24^ and prostate cancer,^8^ were prospectively measured for all patients. The exclusion criteria were as follows: no extended (≥10 cores) systematic biopsy performed, pathologic T0 (vanishing tumor phenomenon), and incomplete clinical or pathologic data on review. A total of 793 patients fulfilled these criteria and were enrolled. Two hundred and seventy‐two men who were confirmed negative for malignancy, and 54 men who were initially diagnosed with prostate cancer affecting the non‐regional lymph node (M1a), or that had distantly metastasized (M1b or M1c stage), were used as reference groups for the comparison of clinical parameters, including serum IGF‐1 level.

### 2.2 Measurement of serum IGF‐1, IGFBP‐3, and testosterone levels

For the measurement of glucose, IGF‐1, IGFBP‐3, and testosterone level, serum samples were obtained by cubital venipuncture in fasting patients between 06:00 and 08:00, immediately before RP. In the reference groups, serum IGF‐1 levels were measured on the date of prostatic biopsy in the fasting state. The following hormones were quantified on the date of sampling using the following commercially available kits: IGF‐1 (immunoradiometric assay A15729, Immunotech, Prague, Czech Republic), IGFBP‐3 (immunoradiometric assay CL‐BC1014, Immunodiagnostic Systems, Boldon, UK), and testosterone (radioimmunoassay TESTO‐CT2, Cisbio Bioassays, Codolet, France). Free and bioavailable testosterone levels were calculated from the measured levels of testosterone, albumin, and SHBG.^25^

### 2.3 Data collection and definitions

The following data were collected from the electronic medical record system: medical history, age, height, body mass, total prostatic volume (TPV) measured by transrectal ultrasonography, serum PSA level, biopsy GS, clinical tumor‐node‐metastasis (TNM) staging, surgical GS, and pathologic TNM staging. All biopsy and surgical GSs were determined by specialized genitourinary pathologists, who were blinded to the serum IGF‐1, IGFBP‐3, and testosterone levels. The revised (2005) criteria for GS were adopted,^26^ and TNM staging was performed according to the revised recommendations of the American Joint Cancer Committee in 2010.^27^

### 2.4 Statistical analysis

Clinical parameters, including serum levels of IGF‐1, IGFBP‐3, testosterone, and free and bioavailable testosterone, were compared according to surgical GS (classified into low [GS ≤ 6], intermediate [GS = 7], and high [GS ≥ 8]). Clinical characteristics were also compared between prostate cancer patients and reference groups. Unconditional logistic regression analysis was performed to estimate odds ratios (ORs) and 95% confidence intervals (CIs) for localized prostate cancer, high surgical GS, advanced pathologic stage (≥pT3), and metastatic (M1) disease, according to serum IGF‐1 quartile. The risk of high surgical GS by quartile of bioavailable testosterone level was also assessed. Using spline curve analysis, the optimal cut‐off values for serum IGF‐1 and bioavailable testosterone to discriminate high surgical GS were estimated, and the risks of high surgical GS for patients in four categories (normal/low IGF‐1 and normal/low bioavailable testosterone) were assessed. In prostate cancer patients with biopsy GS ≤ 3 + 4, the actual prevalences of upgrading to high surgical GS were also compared according to patient bioavailable testosterone and IGF‐1 levels. Binary logistic regression analyses were performed in the entire prostate cancer cohort to identify the independent preoperative predictive factors for high surgical GS. Continuous parameters were compared between groups using one‐way analysis of variance (ANOVA) test or Student's *t* test, and categorical parameters were compared using the *χ* ^2^ test. All tests were two‐tailed, and statistical significance was accepted when *P *<* *0.05. All statistical analyses were performed using SPSS^®^ version 21.0 (IBM, Chicago, IL, USA) or R version 3.2.0 (R Development Core Team, https://www.r-project.org/).

## 3 RESULTS

### 3.1 Correlation between serum IGF‐1 and prostate cancer risk

Patients with prostate cancer had higher serum IGF‐1 (143.8 vs 118.9 ng/mL) and PSA (8.6 vs 5.6 ng/mL) levels than men without prostate cancer (all *P *<* *0.001; Table S1). High serum IGF‐1 was associated with a high risk of localized prostate cancer (highest vs lowest quartile, OR = 3.35; 95% CI, 2.40‐4.69, *P*_trend_ < 0.001; Table 1).

**Table 1:** Odds ratios for prostate cancer, according to the quartiles of serum insulin‐like growth factor‐1 levels in men without cancer

IGF‐1 (ng/mL)	Quartile								Continuous variable	*P*‐value
	1Q		2Q		3Q		4Q			
	Ca/Co	OR (95% CI)	Ca/Co	OR (95% CI)	Ca/Co	OR (95% CI)	Ca/Co	OR (95% CI)		
	≤89.5		89.5‐112.0		112.0‐144.0		>144.0		per 100 ng/mL	
All tumors	104/68	1.00 (ref)	109/70	1.02 (0.66‐1.56)	210/67	2.05 (1.36‐3.09)	370/67	3.61 (2.41‐5.39)	3.35 (2.40‐4.69)	<0.001*
Surgical GS										
GS ≤ 7	81/68	1.00 (ref)	81/70	0.97 (0.62‐1.53)	175/67	2.19 (1.43‐3.36)	324/67	4.06 (2.68‐6.15)	3.70 (2.62‐5.23)	<0.001*
GS ≥ 8	23/68	1.00 (ref)	28/70	1.18 (0.62‐2.25)	35/67	1.54 (0.83‐2.89)	46/67	2.03 (1.11‐3.71)	2.05 (1.27‐3.30)	0.003*
Biopsy GS										
GS ≤ 7	72/68	1.00 (ref)	76/70	1.03 (0.65‐1.63)	166/67	2.34 (1.51‐3.62)	283/67	3.99 (2.60‐6.10)	3.63 (2.55‐5.16)	<0.001*
GS ≥ 8	32/68	1.00 (ref)	33/70	1.00 (0.56‐1.81)	44/67	1.40 (0.79‐2.46)	87/67	2.76 (1.63‐4.68)	2.71 (1.76‐4.16)	<0.001*
Tumor stage										
≤pT2	68/68	1.00 (ref)	65/70	0.93 (0.58‐1.50)	128/67	1.91 (1.22‐2.99)	235/67	3.51 (2.28‐5.40)	3.51 (2.43‐5.05)	<0.001*
≥pT3	36/68	1.00 (ref)	44/70	1.19 (0.68‐2.06)	82/67	2.31 (1.38‐3.88)	135/67	3.81 (2.31‐6.27)	3.20 (2.16‐4.73)	<0.001*

Ca/Co, case per control ratio; CI, confidence interval; GS, Gleason score.; IGF, insulin‐like growth factor; OR, odds ratio.

* *P* < 0.05.

### 3.2 Association between serum IGF‐1 level and surgical tumor grade

Among the 793 men with localized prostate cancer who underwent RP, low, intermediate, and high surgical GSs was observed in 159 (20.1%), 502 (63.3%), and 132 (16.6%) patients, respectively (Table 2). Serum levels of IGF‐1 (*P *=* *0.006) and IGFBP‐3 (*P *=* *0.048) tended to be lower, while the serum PSA level (*P *<* *0.001) tended to be higher, in patients with higher surgical GS (Table 2). Serum testosterone, free testosterone, and bioavailable testosterone levels were not significantly different among the three GS groups (*P* range, 0.210‐0.852; Table 2). When men with localized prostate cancer were analyzed in quartiles, high serum IGF‐1 level was associated with a low risk of high surgical GS (OR = 0.464; *P*_trend_ = 0.006; Table 3), whereas serum IGF‐1 showed no significant association with the risk of advanced pathologic stage (*P*_trend_ = 0.911; Table S2).

**Table 2:** Comparison of clinical and pathologic characteristics, according to the surgical Gleason score

	Surgical GS ≤ 6 (A)	Surgical GS = 7 (B)	Surgical GS ≥ 8 (C)	*P*‐value^a^			
				All	(A)‐(B)	(B)‐(C)	(A)‐(C)
Number of patients	159	502	132	—	—	—	—
Demographics							
Age (y)	63.4 (±6.9)	65.4 (±7.0)	66.5 (±6.7)	<0.001*	0.003*	0.242	<0.001*
BMI (kg/m^2^)	24.7 (±2.8)	24.7 (±2.7)	24.7 (±3.1)	0.984	—	—	—
Comorbidity							
Hypertension	67 (42.1%)	223 (44.4%)	61 (46.2%)	0.779	—	—	—
Diabetes mellitus	25 (15.7%)	89 (17.7%)	26 (19.7%)	0.674	—	—	—
Blood tests							
PSA (ng/mL)	5.9 (±3.6)	8.4 (±6.4)	12.5 (±12.4)	<0.001*	0.001*	<0.001*	<0.001*
IGF‐1 (ng/mL)	151.7 (±49.8)	144.1 (±50.0)	132.9 (±46.4)	0.006*	0.210	0.055	0.004*
IGFBP‐3 (ng/mL)	2121.2 (±456.9)	2056.5 (±499.7)	1981.2 (±440.9)	0.048*	0.304	0.247	0.037*
Testosterone (ng/mL)	4.4 (±1.3)	4.4 (±1.3)	4.3 (±1.3)	0.852	—	—	—
Free testosterone (pg/mL)	58.6 (±17.1)	56.9 (±18.2)	54.8 (±18.9)	0.203	—	—	—
Bioavailable testosterone (ng/mL)	1.3 (±0.4)	1.3 (±0.4)	1.3 (±0.4)	0.210	—	—	—
SHBG (nmol/L)	64.5 (±22.3)	69.1 (±26.1)	74.4 (±27.5)	0.060	—	—	—
Glucose (mg/dL)	110.3 (±30.4)	113.5 (±34.6)	120.0 (±48.6)	0.071	—	—	—
Total prostate volume (mL)	41.6 (±21.2)	33.1 (±12.3)	36.4 (±16.8)	<0.001*	<0.001*	0.061	0.012*
Pathologic findings							
Percentage of positive cores (%)	18.7 (±15.2)	32.2 (±22.2)	43.4 (±27.8)	<0.001*	<0.001*	<0.001*	<0.001*
Percentage tumor volume (%)	5.6 (±5.7)	14.9 (±15.1)	29.1 (±26.7)	<0.001*	<0.001*	<0.001*	<0.001*
Pathologic T stage							
≤pT2	147 (92.5%)	310 (61.8%)	39 (29.5%)	<0.001*	<0.001*	<0.001*	<0.001*
≥pT3	12 (7.5%)	192 (38.2%)	93 (70.5%)				
Nodal involvements (pN1)	0 (0.0%)	9 (1.8%)	17 (12.9%)	<0.001*	0.089	<0.001*	<0.001*

BMI, body mass index; GS, Gleason score; IGF, insulin‐like growth factor; IGFBP, IGF binding protein; PSA, prostate‐specific antigen; SHBG, sex hormone‐binding globulin.

a One‐way analysis of variance test (continuous variables) and *χ* ^2^ test (categorical variables).

* *P *< 0.05.

**Table 3:** Odds ratios for high surgical Gleason score (≥8), according to the quartiles of serum insulin‐like growth factor‐1 levels of prostate cancer patients

	Quartile				Continuous variable	*P*‐value
	1Q	2Q	3Q	4Q		
IGF‐1 (ng/mL)	≤110.0	110.0‐141.0	141.0‐172.0	>172.0	per 100 ng/mL	
Crude	1.0 (ref)	0.728 (0.443‐1.196)	0.623 (0.374‐1.038)	0.464 (0.268‐0.801)	0.568 (0.379‐0.852)	0.006*
Age adjusted	1.0 (ref)	0.770 (0.466‐1.273)	0.675 (0.401‐1.137)	0.536 (0.301‐0.956)	0.637 (0.415‐0.978)	0.039*
IGFBP‐3 (ng/mL)	≤1743.5	1743.5‐1995.0	1995.0‐2300.0	>2300.0	per 100 ng/mL	
Crude	1.0 (ref)	0.810 (0.486‐1.352)	0.752 (0.448‐1.264)	0.704 (0.415‐1.192)	0.959 (0.921‐1.000)	0.049*
Age adjusted	1.0 (ref)	0.844 (0.505‐1.413)	0.825 (0.487‐1.398)	0.829 (0.478‐1.437)	0.971 (0.930‐1.013)	0.171

IGF, insulin‐like growth factor; IGFBP, IGF binding protein.

* *P* < 0.05.

### 3.3 Associations among serum levels of IGF‐1, testosterone, and bioavailable testosterone

**Figure 1:**
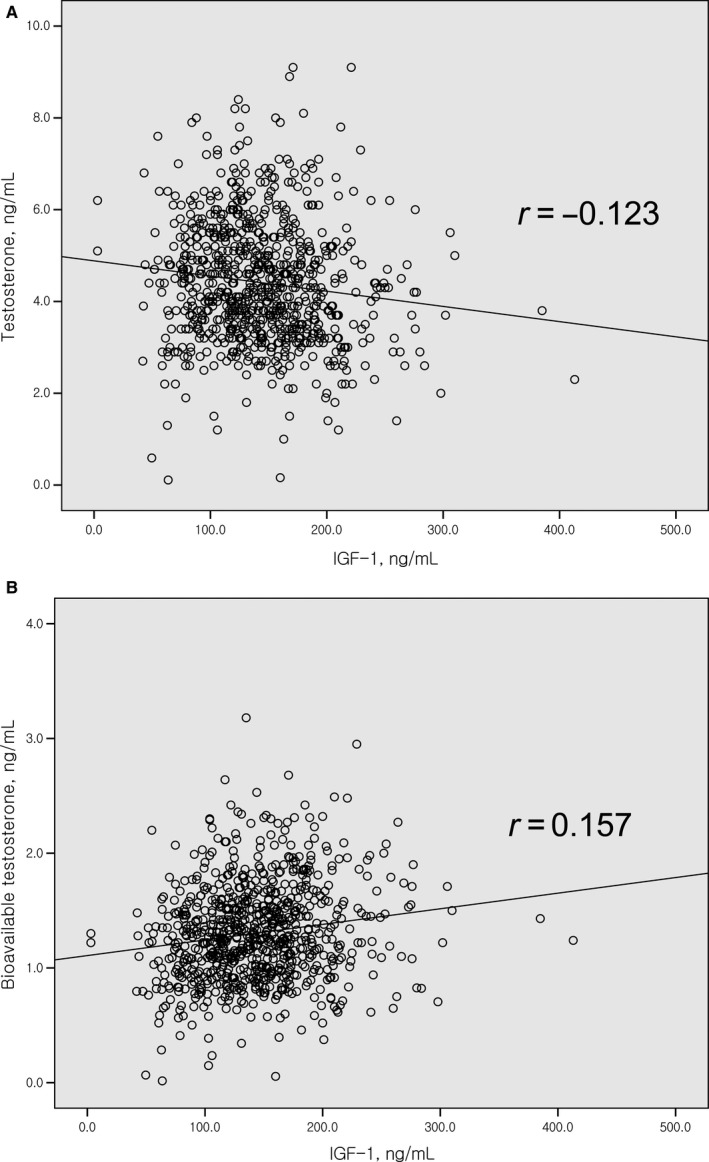
Relationship between serum levels of testosterone and insulin‐like growth factor‐1. Serum total testosterone was inversely correlated with serum IGF‐1 (A; *r* = −0.123, *P* < 0.001), while the level of bioavailable testosterone was positively correlated (B; *r* = 0.157, *P* < 0.001)

Among the 793 men with localized prostate cancer, associations among serum IGF‐1, testosterone, and bioavailable testosterone were assessed (Figure 1). Serum testosterone level was inversely correlated with serum IGF‐1 level (*r *=* *−0.123, *P *<* *0.001; Figure 1A), while serum bioavailable testosterone level was positively correlated with serum IGF‐1 (*r *=* *0.157, *P *<* *0.001; Figure 1B). High serum bioavailable testosterone level tended to be associated with a low risk of high surgical GS without a statistical significance (OR = 0.702; *P*_trend_ = 0.173; Table 4), as did serum IGF‐1.

**Table 4:** Associations among the serum levels of bioavailable testosterone, insulin‐like growth factor‐1, and high surgical Gleason score (≥8)

	Quartile				Continuous variable	*P*‐value
	1Q	2Q	3Q	4Q		
Bioavailable T (ng/mL)	≤1.00	1.00‐1.27	1.27‐1.56	>1.56	per 1 ng/mL	
Crude	1.0 (ref)	0.459 (0.266‐0.793)	0.689 (0.417‐1.140)	0.702 (0.424‐1.162)	0.734 (0.470‐1.146)	0.173
IGF‐1 adjusted	1.0 (ref)	0.478 (0.276‐0.829)	0.705 (0.425‐1.169)	0.781 (0.468‐1.304)	0.809 (0.514‐1.273)	0.359
IGF‐1 (ng/mL)	≤110.0	110.0‐141.0	141.0‐172.0	>172.0	per 100 ng/mL	
Crude	1.0 (ref)	0.728 (0.443‐1.196)	0.623 (0.374‐1.038)	0.464 (0.268‐0.801)	0.568 (0.379‐0.852)	0.006*
Bioavailable T adjusted	1.0 (ref)	0.748 (0.454‐1.234)	0.640 (0.383‐1.070)	0.484 (0.278‐0.843)	0.587 (0.390‐0.884)	0.011*

IGF, insulin‐like growth factor; T, testosterone.

* *P *< 0.05.

### 3.4 Relationships between the risk of high surgical GS and serum IGF‐1 or bioavailable testosterone level

**Figure 2:**
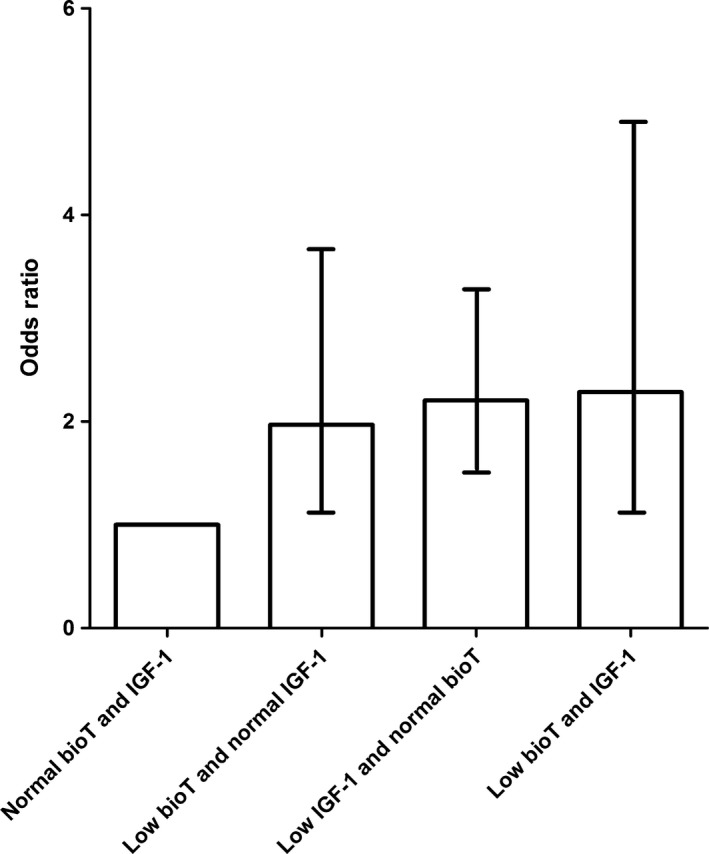
Odds ratio (OR) and 95% confidence interval (CI) for high surgical Gleason score (≥8), according to serum levels of bioavailable testosterone (bioT) and insulin‐like growth factor (IGF)‐1. The OR for high surgical Gleason score was the highest (OR = 2.285; 95% CI, 1.066‐4.897) in patients with low bioT (≤0.85 ng/mL) and low IGF‐1 (≤116.0 ng/mL), followed by when one of bioT (OR = 1.968; 95% CI, 1.045‐3.704) or IGF‐1 (OR = 2.205; 95% CI, 1.442‐3.371) was low

The optimal cut‐off values for serum IGF‐1 and bioavailable testosterone to discriminate high surgical GS were shown to be 116.0 and 0.85 ng/mL, respectively (Figure S1). When the men with prostate cancer were divided into four categories according to these cut‐off values (Figure 2), the risk of high surgical GS was highest (OR = 2.285; 95% CI, 1.066‐4.897) in patients with low bioavailable testosterone (≤0.85 ng/mL) and low IGF‐1 (≤116.0 ng/mL), and was less when the level of either bioavailable testosterone (OR = 1.968; 95% CI, 1.045‐3.704) or IGF‐1 was low (OR = 2.205; 95% CI, 1.442‐3.371; Figure 2). Among patients with biopsy GS ≤ 3 + 4 (*n *=* *460), the prevalence of upgrading to high surgical GS was significantly higher in patients who had low serum bioavailable testosterone (≤0.85 ng/mL; 9.3% or 5/54) or low IGF‐1 (≤116.0 ng/mL; 9.9% or 12/121) than in patients who had normal bioavailable testosterone and IGF‐1 levels (2.6% or 8/307).

### 3.5 Associations of prostate size, tumor volume, and serum IGF‐1 or bioavailable testosterone level

In patients with localized prostate cancer (*n *=* *793), prostate volume, percentage of positive core, and tumor volume were not associated with either serum IGF‐1 level or bioavailable testosterone level (*P* range, 0.284‐0.833; Table S4). Moreover, after adjustment for preoperative parameters, including prostate volume or the percentage of positive core, a low serum IGF‐1 level was an independent predictor of high surgical GS (OR = 1.012; *P *=* *0.018; Table 5). These results suggest that the inverse association between serum IGF‐1 and high surgical GS is not driven by the effects of altering prostate or tumor volume.

**Table 5:** Binary logistic regression analysis for high surgical Gleason score (≥8)

	Univariate^a^		Multivariate^a^	
	OR (95% CI)	*P*‐value	OR (95% CI)	*P*‐value
Age (y)	1.034 (1.006‐1.063)	0.018	1.005 (0.970‐1.041)	0.791
Height (cm)	0.986 (0.952‐1.020)	0.402	1.002 (0.955‐1.051)	0.929
Weight (kg)	0.997 (0.976‐1.019)	0.784	0.995 (0.967‐1.024)	0.734
PSA (ng/mL)	1.071 (1.046‐1.096)	<0.001*	1.035 (1.005‐1.066)	0.022*
Prostate volume (mL)	1.005 (0.994‐1.017)	0.370	1.014 (1.001‐1.028)	0.042*
Clinical T stage (≥cT2 vs cTx, ≤cT1)	1.360 (0.927‐1.994)	0.115	0.948 (0.591‐1.520)	0.824
Biopsy GS (≥8 vs <8)	14.236 (9.215‐21.992)	<0.001*	11.682 (7.364‐18.532)	<0.001*
Percentage of positive core (%)	1.024 (1.016‐1.031)	<0.001*	1.012 (1.002‐1.022)	0.020*
IGFBP‐3 (ng/mL)	0.999 (0.999‐1.000)	0.049*	1.000 (0.999‐1.001)	0.991
Bioavailable testosterone ≤ 0.85 (ng/mL)	1.583 (0.969‐2.588)	0.068	1.406 (0.771‐2.567)	0.266
IGF‐1 ≤ 116.0 (ng/mL)	2.000 (1.362‐2.938)	<0.001*	1.790 (1.076‐2.979)	0.025*

CI, confidence interval; GS, Gleason score; IGF, insulin‐like growth factor; IGFBP, IGF binding protein; OR, odds ratio; PSA, prostate‐specific antigen.

a Binary logistic regression analysis.

* *P* < 0.05.

### 3.6 Association between serum IGF‐1 level and distant metastasis

Patients with metastatic prostate cancer had lower serum IGF‐1 than those with localized disease (128.0 vs 143.8 ng/mL, *P *=* *0.023). However, almost all the patients with metastatic disease (90.7%) had high biopsy GS (Table S5). In patients with high biopsy GS (196 with localized and 49 with metastatic disease), serum IGF‐1 levels for those with localized and for those with metastatic disease were not significantly different (139.6 vs 130.8 ng/mL, *P *=* *0.260). Similarly, among the 793 men with localized prostate cancer and 54 with metastatic disease, the inverse association between serum IGF‐1 level and risk of metastatic disease was no longer significant after adjustment for biopsy GS (*P*_trend_ = 0.079; Table S6). These results suggest that the inverse association between serum IGF‐1 and metastatic disease is mainly driven by tumor grade, rather than by the extent of the disease.

## 4 DISCUSSION

### 4.1 Previous clinical studies of the relationship between serum IGF‐1 and tumor grade

Previous clinical studies investigated the association between serum IGF‐1 levels and tumor aggressiveness, but their results were not consistent with the epidemiologic data^28^, ^29^, ^30^ due to small sample size, such that they had insufficient statistical power to discriminate tumor grade. All three of the quoted studies demonstrated trends toward lower IGF‐1 level in high‐grade disease, but these fell short of statistical significance.^28^, ^29^, ^30^ Of these previous studies, the one with the largest sample size (*n *=* *242) used a GS based on prostatic biopsy, rather than surgical pathology.^30^ This is significant because a low or intermediate biopsy GS can be associated with a high surgical GS, due to the fact that biopsy scoring underestimates the extent of dedifferentiation.^31^

In our cohort, a mismatch between biopsy and surgical GS was observed in 45.3% of patients (data not shown). This discrepancy was caused by the innate limitation of prostate biopsy: that the characteristics of a biopsy are never fully representative of the characteristics of the entire tumor. The present study had a robust sample size (*n *=* *793) and clearly demonstrated a lower risk of high surgical GS in patients with high serum IGF‐1 level (highest vs lowest quartile, OR = 0.494; *P*_trend_ = 0.006; Table 3), whereas the risk of prostate cancer itself was positively associated with serum IGF‐1 level (OR = 3.35, *P*_trend_ < 0.001; Table 1). These findings are in agreement with those of previous epidemiologic studies, such as the HPFS,^10^, ^11^, ^12^ PHS,^14^ and EPIC^15^ studies.

### 4.2 Associations among serum levels of IGF‐1 and testosterone, and tumor grade

We hypothesized that the inverse association between serum IGF‐1 level and tumor grade is due to the autonomy of high‐grade prostate cancer, similar to the inverse relationship between testosterone availability and high‐grade disease.^19^, ^20^, ^21^, ^22^, ^23^ To test this hypothesis, we assessed the relationship between serum IGF‐1 levels and testosterone levels, and found that the serum testosterone level itself is inversely proportional to the IGF‐1 level (*r *=* *−0.123; Figure 1A), but the bioavailable testosterone level is positively correlated with IGF‐1 (*r *=* *0.157; Figure 1B).

High serum bioavailable testosterone tended to be associated with a lower risk of high surgical GS without a statistical significance (OR = 0.702; *P *=* *0.173; Table 4), as did serum IGF‐1. This finding is consistent with that of the previous study by Leon et al.,^23^ which showed that significantly higher rates of surgical intermediate‐ or high‐grade (GS ≥7) disease were observed in patients with low bioavailable testosterone (≤1.5 ng/mL; 44.3 vs 33.1%; *P *<* *0.001).^23^ Many other recent studies support the contention that high availability of testosterone is associated with a lower risk of high‐grade disease.^19^, ^20^, ^21^, ^22^, ^23^

Little is known about whether serum IGF‐1 levels affect the availability of testosterone. Our current data show that serum IGF‐1 was inversely associated with SHBG (*r *=* *−0.302, *P *<* *0.001; data not shown), suggesting that lower IGF‐1 levels might induce an increase in SHBG level, which could limit the bioavailability of serum testosterone. Moreover, bioavailable testosterone enhances the action of growth hormone (GH),^32^ and it was recently reported that the administration of exogenous testosterone to older men increases serum IGF‐1 *via* the GH pathway.^33^ Because this study was cross‐sectional, we could not determine which of serum IGF‐1 and bioavailable testosterone was the primary determinant. However, it confirms that these two hormones are co‐regulated and suggests that they have a similar tendency to tumorigenesis in prostate cancer. These findings are consistent with our hypothesis that the inverse association between serum IGF‐1 or testosterone and tumor grade is caused by the autonomy of high‐grade prostate cancer.

The lack of a relationship between high‐grade disease and IGF‐1 has also been implied in other reports.^34^ The PI3K/AKT pathway is inhibited by phosphatase and tensin homolog (PTEN), which is a tumor suppressor.^35^ A previous study reported that complete loss of the PTEN gene is more frequent in high‐grade prostate cancer,^36^ in which, the PI3K/AKT pathway is constitutively active and requires minimal activation by IGF‐1 or other hormones. In men of the HPFS^10^, ^11^, ^12^ and PHS^14^ cohorts, loss of PTEN was significantly associated with higher IGF1R expression (*P *=* *0.03).^34^ These findings suggest that high‐grade prostate cancer requires minimal activation by IGF‐1, and that the IGF‐1 threshold level for tumorigenesis might be lower.

### 4.3 Clinical implications

**Figure 3:**
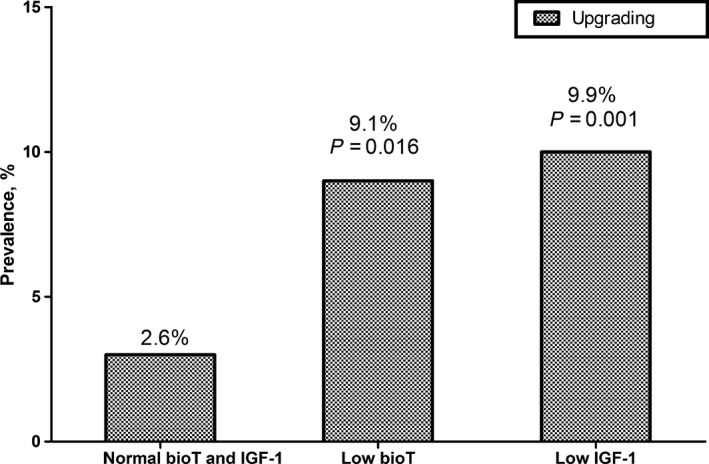
Prevalence of upgrading to high surgical Gleason score (≥8) among patients with biopsy Gleason score ≤ 3 + 4, according to serum levels of bioavailable testosterone (bioT) and insulin‐like growth factor (IGF)‐1. *n *=* *460. The actual prevalence of upgrading was significantly higher in patients who had a low bioT (≤0.85 ng/mL; 9.3% or 5/54) or low IGF‐1 level (≤116.0 ng/mL; 9.9% or 12/121) than in patients who had normal bioT and IGF‐1 levels (2.6% or 8/307)

Serum IGF‐1 and bioavailable testosterone levels were inversely correlated with high surgical GS, and the optimal cut‐off values for serum IGF‐1 and bioavailable testosterone were 116.0 ng/mL and 0.85 ng/mL, respectively (Figure S1). When patients with localized prostate cancer were classified into four categories using these cut‐off values, the probability of high surgical GS was greatest when the levels of the two markers were simultaneously low (OR = 2.285), and less when one of bioavailable testosterone (OR = 1.968) or IGF‐1 (OR = 2.205) was low (Figure 2). Among patients with biopsy GS ≤ 3 + 4, the prevalence of upgrading to high surgical GS was significantly higher in patients with low serum bioavailable testosterone or IGF‐1 than in patients who had normal levels of each (9.1% vs 9.9 vs 2.6%, respectively; Figure 3).

To confirm serum IGF‐1 level as an independent predictor of high surgical GS, multivariate analysis was performed (Table 5). As expected, high biopsy GS was the strongest predictor of high surgical GS (OR = 14.236; *P *<* *0.001). However, low serum IGF‐1 level (≤116.0 ng/mL) was also an independent predictor of high surgical GS (OR = 1.790; *P *=* *0.025). These findings suggest that serum IGF‐1 level may be useful for the prediction of hidden high‐grade disease, which presents as a clinically indolent tumor on prostate biopsy. In patients with low biopsy GS in the clinical setting, there are few markers available for the prediction of occult high surgical GS disease. Therefore, we recommend measuring serum IGF‐1 to rule out occult high surgical GS in patients with low biopsy GS. We also suggest serum a IGF‐1 level of 116.0 ng/mL, as the cut‐off values to be used.

### 4.4 Limitations of the current study

First, the actual oncological outcomes could not be related to the preoperative serum IGF‐1 level because of the relatively short duration of follow‐up. However, the surgical GS is one of the strongest prognostic factors in prostate cancer.^37^ Recently, an inverse association was reported between serum IGF‐1 and cancer‐specific mortality, but this was not statistically significant (*P*_trend_ = 0.08).^38^ Second, because all of our study subjects were Asian, the generalizability of our findings is limited. There may well be inter‐racial heterogeneity in tumor grades or serum levels of IGF‐1, IGFBP‐3, or testosterone, and therefore external validation of our findings using a larger cohort is warranted.

## 5 CONCLUSIONS

Low preoperative serum IGF‐1 levels were associated with a greater risk of high surgical GS. Serum IGF‐1 levels were significantly correlated with serum bioavailable testosterone levels. Low levels of IGF‐1 and bioavailable testosterone were similarly associated with high‐grade disease. These inverse associations suggest that high‐grade prostate cancer develops independently of the concentrations of these two substances. In patients with low or intermediate biopsy GS, upgrading of surgical GS was more frequent when the serum IGF‐1 level was low. Therefore, serum IGF‐1 may represent a valuable marker of surgical GS.

## CONFLICT OF INTEREST

The authors declare no potential conflict of interests.

## References

[1] Cohen P , Peehl DM , Lamson G , Rosenfeld RG . Insulin‐like growth factors (IGFs), IGF receptors, and IGF‐binding proteins in primary cultures of prostate epithelial cells. J Clin Endocrinol Metab. 1991;73:401‐407. 1713219 10.1210/jcem-73-2-401

[2] Cohen P , Peehl DM , Rosenfeld RG . The IGF axis in the prostate. Horm Metab Res. 1994;26:81‐84. 8200618 10.1055/s-2007-1000777

[3] Rajah R , Valentinis B , Cohen P . Insulin‐like growth factor (IGF)‐binding protein‐3 induces apoptosis and mediates the effects of transforming growth factor‐beta1 on programmed cell death through a p53‐ and IGF‐independent mechanism. J Biol Chem. 1997;272:12181‐12188. 9115291 10.1074/jbc.272.18.12181

[4] Jones JI , Clemmons DR . Insulin‐like growth factors and their binding proteins: biological actions. Endocr Rev. 1995;16:3‐34. 7758431 10.1210/edrv-16-1-3

[5] Belfiore A , Malaguarnera R . Insulin receptor and cancer. Endocr Relat Cancer. 2011;18:R125‐R147. 21606157 10.1530/ERC-11-0074

[6] Albanes D , Taylor PR . International differences in body height and weight and their relationship to cancer incidence. Nutr Cancer. 1990;14:69‐77. 2367236 10.1080/01635589009514078

[7] Wilding G . Endocrine control of prostate cancer. Cancer Surv. 1995;23:43‐62. 7621473

[8] Kaaks R , Lukanova A , Sommersberg B . Plasma androgens, IGF‐1, body size, and prostate cancer risk: a synthetic review. Prostate Cancer Prostatic Dis. 2000;3:157‐172. 12497092 10.1038/sj.pcan.4500421

[9] Renehan AG , Zwahlen M , Minder C , O'Dwyer ST , Shalet SM , Egger M . Insulin‐like growth factor (IGF)‐I, IGF binding protein‐3, and cancer risk: systematic review and meta‐regression analysis. Lancet. 2004;363:1346‐1353. 15110491 10.1016/S0140-6736(04)16044-3

[10] Platz E , Pollak M , Leitzmann M , Stampfer M , Willett W , Giovannucci E . Plasma insulin‐like growth factor‐1 and binding protein‐3 and subsequent risk of prostate cancer in the PSA era. Cancer Causes Control. 2005;16:255‐262. 15947877 10.1007/s10552-004-3484-8

[11] Nimptsch K , Platz EA , Pollak MN , et al. Plasma insulin‐like growth factor 1 is positively associated with low‐grade prostate cancer in the Health Professionals Follow‐up Study 1993–2004. Int J Cancer. 2011;128:660‐667. 20473871 10.1002/ijc.25381 PMC2948057

[12] Cao Y , Nimptsch K , Shui IM , et al. Prediagnostic plasma IGFBP‐1, IGF‐1 and risk of prostate cancer. Int J Cancer. 2015;136:2418‐2426. 25348852 10.1002/ijc.29295 PMC4360136

[13] Roddam AW , Allen NE , Appleby P , et al. Insulin‐like growth factors, their binding proteins, and prostate cancer risk: analysis of individual patient data from 12 prospective studies. Ann Intern Med. 2008;149:461‐471. 18838726 10.7326/0003-4819-149-7-200810070-00006 PMC2584869

[14] Chan JM , Stampfer MJ , Ma J , et al. Insulin‐like growth factor‐I (IGF‐I) and IGF binding protein‐3 as predictors of advanced‐stage prostate cancer. J Natl Cancer Inst. 2002;94:1099‐1106. 12122101 10.1093/jnci/94.14.1099

[15] Prince A , Allen N , Appleby P , Crowe F , Travis R , Tipper S . Insulin‐like growth factor‐1 concentration and risk of prostate cancer: results from the European Prospective Investigation into Cancer and Nutrition. Cancer Epidemiol Biomarkers Prev. 2012;21:1531‐1541. 22761305 10.1158/1055-9965.EPI-12-0481-T PMC5749609

[16] Huggins C , Hodges CV . Studies on prostatic cancer, I: the effect of castration, of estrogen and of androgen injection on serum phosphatases in metastatic carcinoma of the prostate. Cancer Res. 1941;1:293‐297. 10.3322/canjclin.22.4.232 4625049

[17] Morgentaler A . Testosterone and prostate cancer: an historical perspective on a modern myth. Eur Urol. 2006;50:935‐939. 16875775 10.1016/j.eururo.2006.06.034

[18] Parsons JK , Carter HB , Platz EA , Wright EJ , Landis P , Metter EJ . Serum testosterone and the risk of prostate cancer: potential implications for testosterone therapy. Cancer Epidemiol Biomarkers Prev. 2005;14:2257‐2260. 16172240 10.1158/1055-9965.EPI-04-0715

[19] Yano M , Imamoto T , Suzuki H , Fukasawa S , Kojima S , Komiya A , et al. The clinical potential of pretreatment serum testosterone level to improve the efficiency of prostate cancer screening. Eur Urol. 2007;51:375‐380. 17005316 10.1016/j.eururo.2006.08.047

[20] Schatzl G , Madersbacher S , Thurridl T , Waldmüller J , Kramer G , Haitel A , et al. High‐grade prostate cancer is associated with low serum testosterone levels. Prostate. 2001;47:52‐58. 11304729 10.1002/pros.1046

[21] Hoffman MA , DeWolf WC , Morgentaler A . Is low serum free testosterone a marker for high grade prostate cancer? J Urol. 2000;163:824‐827. 10687985

[22] Banach‐Petrosky W , Jessen WJ , Ouyang X , et al. Prolonged exposure to reduced levels of androgen accelerates prostate cancer progression in Nkx3.1; Pten mutant mice. Cancer Res. 2007;67:9089‐9096. 17909013 10.1158/0008-5472.CAN-07-2887

[23] Leon P , Seisen T , Cussenot O , et al. Low circulating free and bioavailable testosterone levels as predictors of high‐grade tumors in patients undergoing radical prostatectomy for localized prostate cancer. Urol Oncol. 2015;33:384.e21‐27. 10.1016/j.urolonc.2014.11.010 25595576

[24] Rechler MM . Growth inhibition by insulin‐like growth factor (IGF) binding protein‐3‐what's IGF got to do with it? Endocrinology. 1997;138:2645‐2647. 9202199 10.1210/endo.138.7.5355

[25] Vermeulen A , Verdonck L , Kaufman JM . A critical evaluation of simple methods for the estimation of free testosterone in serum. J Clin Endocrinol Metab. 1999;84:3666‐3672. 10523012 10.1210/jcem.84.10.6079

[26] Epstein JI , Allsbrook WCJ , Amin MB , Egevad LL ; ISUP Grading Committee . The 2005 International Society of Urological Pathology (ISUP) consensus conference on Gleason grading of prostatic carcinoma. Am J Surg Pathol. 2005;29:1228‐1242. 16096414 10.1097/01.pas.0000173646.99337.b1

[27] Edge SB , Byrd DR , Compton CC , Fritz AG , Greene FL , Andy TI . American Joint Committee on Cancer: 41. Prostate In: Edge SB, Byrd DR, Compton CC, Fritz AG, Greene FL, Andy TI, eds. AJCC Cancer Staging Manual, 7th ed. New York, NY: Springer; 2010:457‐468.

[28] Correa LL , Neto LV , Lima GA , Gabrich R , de Miranda LC , Gadelha MR . Insulin‐like growth factor (IGF)‐I, IGF binding protein‐3, and prostate cancer: correlation with Gleason score. Int Braz J Urol. 2015;41:110‐115. 25928516 10.1590/S1677-5538.IBJU.2015.01.15 PMC4752063

[29] Hong SK , Han BK , Jeong JS , et al. Serum measurements of testosterone, insulin‐like growth factor 1, and insulin‐like growth factor binding protein‐3 in the diagnosis of prostate cancer among Korean men. Asian J Androl. 2008;10:207‐213. 18097534 10.1111/j.1745-7262.2008.00296.x

[30] Ismail HA , Pollak M , Behlouli H , Tanguay S , Bégin LR , Aprikian AG . Serum insulin‐like growth factor (IGF)‐1 and IGF‐binding protein‐3 do not correlate with Gleason score or quantity of prostate cancer in biopsy samples. BJU Int. 2003;92:699‐702. 14616449 10.1046/j.1464-410x.2003.04084.x

[31] Epstein JI , Sanderson H , Carter HB , Scharfstein DO . Utility of saturation biopsy to predict insignificant cancer at radical prostatectomy. Urology. 2005;66:356‐360. 16040085 10.1016/j.urology.2005.03.002

[32] Birzniece V , Ho KKY . Sex steroids and the GH axis: Implications for the management of hypopituitarism. Best Pract Res Clin Endocrinol Metab. 2017;31:59‐69. 28477733 10.1016/j.beem.2017.03.003

[33] Dias JP , Veldhuis JD , Carlson O , et al. Effects of transdermal testosterone gel or an aromatase inhibitor on serum concentration and pulsatility of growth hormone in older men with age‐related low testosterone. Metabolism. 2017;69:143‐147. 28285644 10.1016/j.metabol.2017.01.025 PMC5950718

[34] Zu K , Martin NE , Fiorentino M , et al. Protein expression of PTEN, insulin‐like growth factor I receptor (IGF‐IR), and lethal prostate cancer: a prospective study. Cancer Epidemiol Biomarkers Prev. 2013;22:1984. 23983239 10.1158/1055-9965.EPI-13-0349 PMC3818474

[35] Carver BS , Chapinski C , Wongvipat J , et al. Reciprocal feedback regulation of PI3K and androgen receptor signaling in PTEN‐deficient prostate cancer. Cancer Cell. 2011;19(5):575‐586. 21575859 10.1016/j.ccr.2011.04.008 PMC3142785

[36] McMenamin ME , Soung P , Perera S , Kaplan I , Loda M , Sellers WR . Loss of PTEN expression in paraffin‐embedded primary prostate cancer correlates with high Gleason score and advanced stage. Cancer Res. 1999;59(17):4291‐4296. 10485474

[37] Egevad L , Granfors T , Karlberg L , Bergh A , Stattin P . Prognostic value of the Gleason score in prostate cancer. BJU Int. 2002;89:538‐542. 11942960 10.1046/j.1464-410x.2002.02669.x

[38] Cao Y , Lindstrom S , Schumacher F , et al. Insulin‐like growth factor pathway genetic polymorphisms, circulating IGF1 and IGFBP3, and prostate cancer survival. J Natl Cancer Inst. 2014;106:dju085. 24824313 10.1093/jnci/dju085 PMC4081624

