# Supplementary figures and images for: Association between serum levels of insulin‐like growth factor‐1, bioavailable testosterone, and pathologic Gleason score
Source: Cancer Med. 2018 Jul 10;7(8):4170–80. doi: 10.1002/cam4.1681 (PMC6089192; doi:10.1002/cam4.1681)

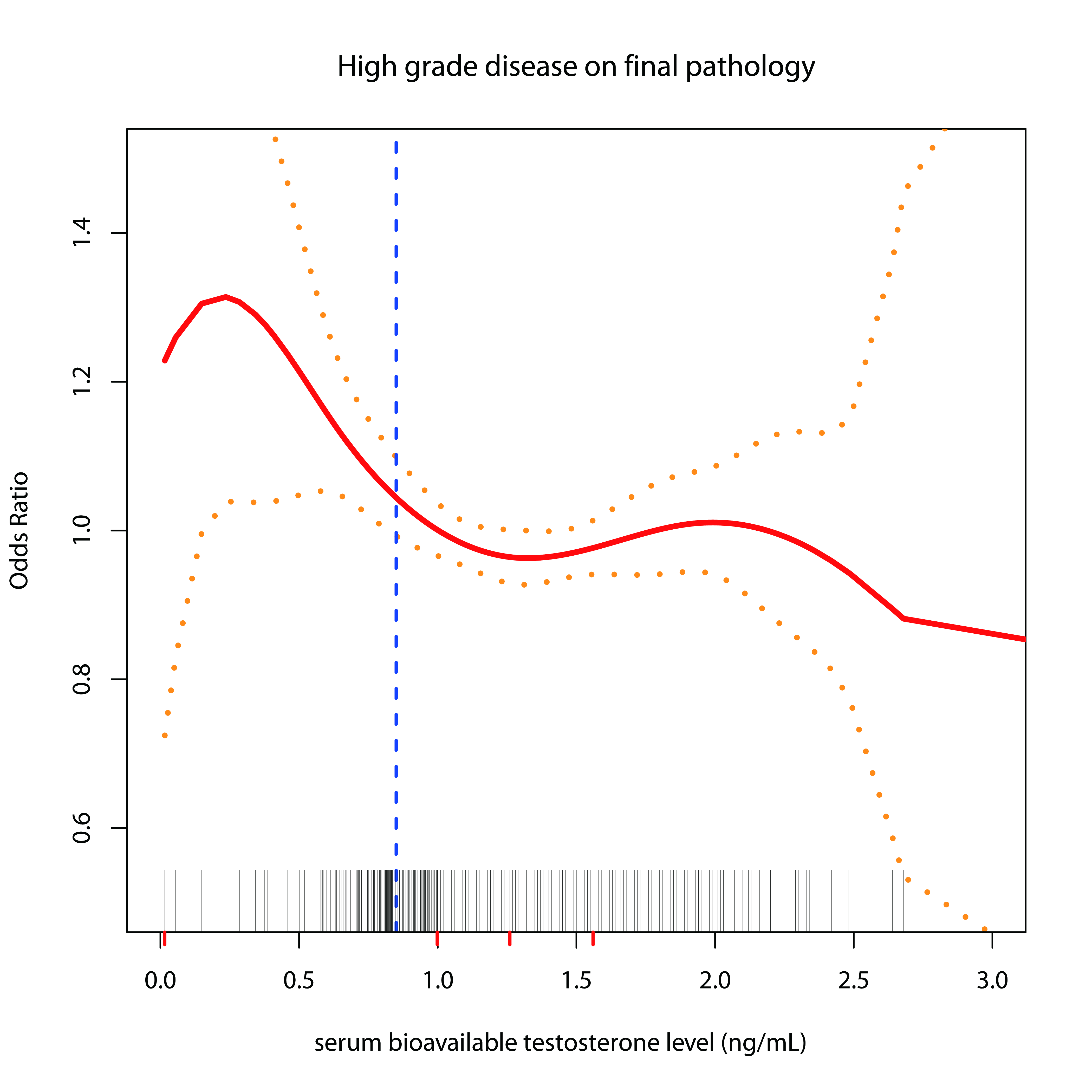

Supplement: Supplementary file 1 [file CAM4-7-4170-s001.tif]

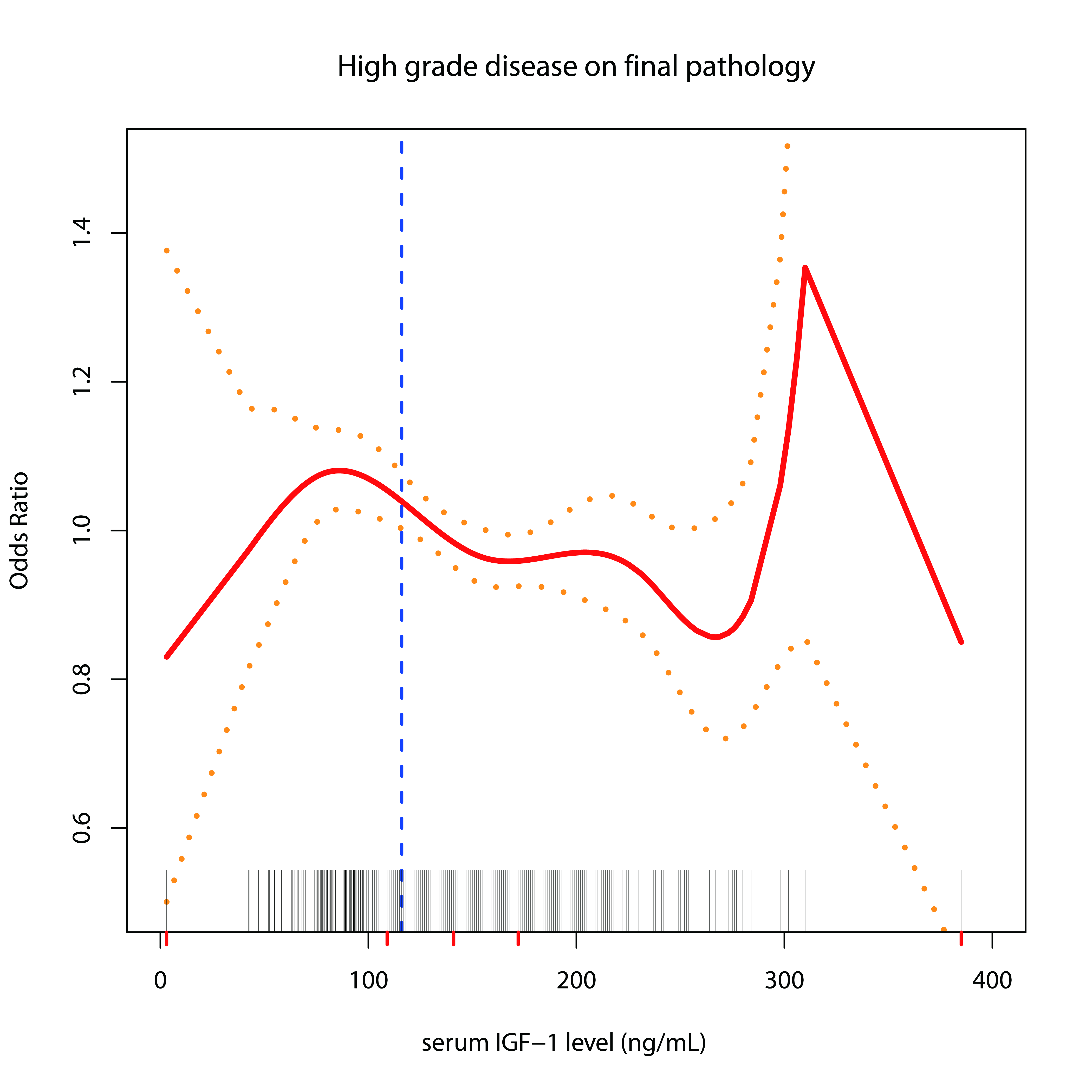

Supplement: Supplementary file 2 [file CAM4-7-4170-s002.tif]
