# Supplementary material for: Association between serum levels of insulin‐like growth factor‐1, bioavailable testosterone, and pathologic Gleason score
Source: Cancer Med. 2018 Jul 10;7(8):4170–80. doi: 10.1002/cam4.1681 (PMC6089192; doi:10.1002/cam4.1681)
Supplement: Supplementary file 3 [file CAM4-7-4170-s003.docx]

**Supporting Table 1.** Comparison of clinical characteristics between men with or without prostate cancer

|  | **With prostate cancer** | **Without prostate cancer** | ***p*-value**† |  |
| --- | --- | --- | --- | --- |
| **Number of patients** | | 793 | 272 | – |
| **Patients characteristics** | |  |  |  |
| Age (years) | | 65.2 (±7.0) | 63.8 (±8.4) | 0.009* |
| BMI (kg/m^2^) | | 24.7 (±2.8) | 24.7 (±3.1) | 0.914 |
| Comorbidity | |  |  |  |
| Hypertension | | 351 (44.3%) | 114 (41.9%) | 0.500 |
| Diabetes mellitus | | 140 (17.7%) | 39 (14.3%) | 0.207 |
| PSA (ng/mL) | | 8.6 (±7.6) | 5.6 (±5.4) | <0.001* |
| IGF-1 (ng/mL) | | 143.8 (±49.7) | 118.9 (±42.4) | <0.001* |
| Total prostate volume (mL) | | 35.3 (±15.6) | 45.6 (±22.3) | <0.001* |

BMI, body mass index; PSA, prostate specific antigen; IGF, insulin-like growth factor

†, Student’s *t-*test (continuous variables) and *χ*^2^ test (categorical variables); *, *p* <0.05
