# Supplementary material for: Association between serum levels of insulin‐like growth factor‐1, bioavailable testosterone, and pathologic Gleason score
Source: Cancer Med. 2018 Jul 10;7(8):4170–80. doi: 10.1002/cam4.1681 (PMC6089192; doi:10.1002/cam4.1681)
Supplement: Supplementary file 4 [file CAM4-7-4170-s004.docx]

**Supporting Table 2.** Odds ratios for advanced disease (≥ pT3), according to the quartiles of serum insulin-like growth factor-1 levels of prostate cancer patients

|  | **Quartile** | | | | **Continuous variable** | ***p*-value** |  |
| --- | --- | --- | --- | --- | --- | --- | --- |
|  | **1Q** | **2Q** | **3Q** | **4Q** |  |  |  |
| **IGF-1 (ng/mL)** | | ≤110.0 | 110.0–141.0 | 141.0–172.0 | >172.0 | per 100 ng/mL |  |
| Crude | | 1.0 (ref) | 0.998  (0.666–1.495) | 0.990  (0.661–1.482) | 0.902  (0.600–1.355) | 0.984  (0.736–1.314) | 0.911 |
| Age adjusted | | 1.0 (ref) | 1.099  (0.728–1.657) | 1.134  (0.749–1.717) | 1.154  (0.746–1.783) | 1.190  (0.872–1.624) | 0.272 |
| **IGFBP-3 (ng/mL)** | | ≤1743.5 | 1743.5–1995.0 | 1995.0–2300.0 | >2300.0 | per 100 ng/mL |  |
| Crude | | 1.0 (ref) | 0.817  (0.544–1.228) | 0.971  (0.649–1.452) | 0.830  (0.552–1.248) | 0.984  (0.955–1.014) | 0.296 |
| Age adjusted | | 1.0 (ref) | 0.856  (0.568–1.291) | 1.084  (0.719–1.635) | 1.007  (0.658–1.543) | 0.998  (0.967–1.029) | 0.884 |

IGF, insulin-like growth factor; IGFBP, IGF binding protein; *, *p* <0.05
