# Supplementary material for: Association between serum levels of insulin‐like growth factor‐1, bioavailable testosterone, and pathologic Gleason score
Source: Cancer Med. 2018 Jul 10;7(8):4170–80. doi: 10.1002/cam4.1681 (PMC6089192; doi:10.1002/cam4.1681)
Supplement: Supplementary file 5 [file CAM4-7-4170-s005.docx]

**Supporting Table 3.** Associations among the serum levels of bioavailable testosterone, insulin-like growth factor-1, and high surgical Gleason score (≥8)

|  | **Quartile** | | | | **Continuous variable** | ***p*-value** |  |
| --- | --- | --- | --- | --- | --- | --- | --- |
|  | **1Q** | **2Q** | **3Q** | **4Q** |  |  |  |
| **Bioavailable T (ng/mL)** | | ≤1.00 | 1.00–1.27 | 1.27–1.56 | >1.56 | per 1 ng/mL |  |
| Crude | | 1.0 (ref) | 0.459  (0.266–0.793) | 0.689  (0.417–1.140) | 0.702  (0.424–1.162) | 0.734  (0.470–1.146) | 0.173 |
| IGF-1 adjusted | | 1.0 (ref) | 0.478  (0.276–0.829) | 0.705  (0.425–1.169) | 0.781  (0.468–1.304) | 0.809  (0.514–1.273) | 0.359 |
| **IGF-1 (ng/mL)** | | ≤110.0 | 110.0–141.0 | 141.0–172.0 | >172.0 | per 100 ng/mL |  |
| Crude | | 1.0 (ref) | 0.728  (0.443–1.196) | 0.623  (0.374–1.038) | 0.464  (0.268–0.801) | 0.568  (0.379–0.852) | 0.006* |
| Bioavailable T adjusted | | 1.0 (ref) | 0.748  (0.454–1.234) | 0.640  (0.383–1.070) | 0.484  (0.278–0.843) | 0.587  (0.390–0.884) | 0.011* |

T, testosterone; IGF, insulin-like growth factor; *, *p* <0.05
