# Supplementary material for: Association between serum levels of insulin‐like growth factor‐1, bioavailable testosterone, and pathologic Gleason score
Source: Cancer Med. 2018 Jul 10;7(8):4170–80. doi: 10.1002/cam4.1681 (PMC6089192; doi:10.1002/cam4.1681)
Supplement: Supplementary file 6 [file CAM4-7-4170-s006.docx]

**Supporting Table 4.** Correlations among serum insulin-like growth factor 1 level, bioavailable testosterone, and other clinical parameters †

|  | Bioavailable T | Prostate volume | PPC | PTV |
| --- | --- | --- | --- | --- |
| IGF-1 (ng/mL) | 0.157** | -0.028 | -0.038 | -0.036 |
| Bioavailable T (ng/mL) | – | -0.011 | -0.008 | -0.034 |
| Prostate volume (mL) | – | – | -0.171** | -0.079* |
| PPC (%) | – | – | – | 0.580** |
| PTV (%) | – | – | – | – |

IGF, insulin-like growth factor; T, testosterone; PPC, percentage of positive core; PTV, percentage of tumor volume

†, Pearson’s correlation analysis; *, *p* <0.05; **, *p* <0.01
