# Supplementary material for: Association between serum levels of insulin‐like growth factor‐1, bioavailable testosterone, and pathologic Gleason score
Source: Cancer Med. 2018 Jul 10;7(8):4170–80. doi: 10.1002/cam4.1681 (PMC6089192; doi:10.1002/cam4.1681)
Supplement: Supplementary file 7 [file CAM4-7-4170-s007.docx]

**Supporting Table 5.** Comparison of clinical characteristics between patients with localized and metastatic prostate cancer

|  | **Localized prostate cancer** | **Metastatic prostate cancer** | ***p*-value**† |  |
| --- | --- | --- | --- | --- |
| **Number of patients** | | 793 | 54 | – |
| **Patients characteristics** | |  |  |  |
| Age (years) | | 65.2 (±7.0) | 69.5 (±6.8) | <0.001* |
| BMI (kg/m^2^) | | 24.7 (±2.8) | 23.4 (±2.6) | 0.003* |
| Comorbidity | |  |  |  |
| Hypertension | | 351 (44.3%) | 31 (57.4%) | 0.060 |
| Diabetes mellitus | | 140 (17.7%) | 12 (22.2%) | 0.397 |
| PSA (ng/mL) | | 8.6 (±7.6) | 177.2 (±298.7) | <0.001* |
| IGF-1 (ng/mL) | | 143.8 (±49.7) | 128.0 (±41.9) | 0.023* |
| Total prostate volume (mL) | | 35.3 (±15.6) | 43.3 (±19.9) | 0.011* |
| Biopsy Gleason score | |  |  |  |
| ≤6 | | 289 (36.4%) | 1 (1.9%) | <0.001* |
| 7 | | 308 (38.8%) | 4 (7.4%) |  |
| ≥8 | | 196 (24.7%) | 49 (90.7%) |  |

BMI, body mass index; PSA, prostate specific antigen; IGF, insulin-like growth factor; †, Student’s *t-*test (continuous variables) and *χ*^2^ test (categorical variables); *, *p* <0.05
