# Supplementary material for: Association between serum levels of insulin‐like growth factor‐1, bioavailable testosterone, and pathologic Gleason score
Source: Cancer Med. 2018 Jul 10;7(8):4170–80. doi: 10.1002/cam4.1681 (PMC6089192; doi:10.1002/cam4.1681)
Supplement: Supplementary file 8 [file CAM4-7-4170-s008.docx]

**Supporting Table 6.** Odds ratios for metastatic disease according to the quartiles of serum insulin-like growth factor-1 level

|  | **Quartile** | | | | **Continuous variable** | ***p*-value** |  |
| --- | --- | --- | --- | --- | --- | --- | --- |
|  | **1Q** | **2Q** | **3Q** | **4Q** |  |  |  |
| **IGF-1 (ng/mL)** | | ≤110.0 | 110.0–141.0 | 141.0–172.0 | >172.0 | per 100 ng/mL |  |
| Crude | | 1.0 (ref) | 0.700  (0.344–1.425) | 0.594  (0.283–1.248) | 0.386  (0.166–0.897) | 0.489  (0.264–0.905) | 0.023* |
| Biopsy Gleason score adjusted | | 1.0 (ref) | 0.868  (0.403–1.872) | 0.702  (0.317–1.554) | 0.498  (0.204–1.215) | 0.552  (0.285–1.072) | 0.079 |

IGF, insulin-like growth factor; *, *p* <0.05
